# Supplementary material for: Basic Health Service Delivery to Vulnerable Populations in Post-Conflict Eastern Congo: Asset Mapping
Source: Healthcare (Basel). 2023 Oct 20;11(20):2778. doi: 10.3390/healthcare11202778 (PMC10606858; doi:10.3390/healthcare11202778)
Supplement: Supplementary file 1 [file healthcare-11-02778-s001.zip › healthcare-2620099-supplementary.pdf]

Supplementary Table S1: Asset map resources sheet

Category of Assets	Abbreviations	Geographic Location	Resources	Collaboration Goals	Organizational Benefit
	Locally known as	*Site in eastern Congo*	*What do they offer?*	*What can you achieve together?*	*How can each group benefit?*
Local associations					
Baraza la Wazee	BARWI	North Kivu	An interethnic organization, an influential partner for the response	Management of intercommunity conflicts	Reduction in atrocities between communities
Youth Counsel		South Kivu, North Kivu, Ituri Province	Protection, education, supervision of youth, and their effective participation in the process of national reconstruction	Promotion of economic development and preservation of Congolese cultural values	Collaboration, friendship, and fraternity with organizations working on youth issues<br>Representation of youth movements
Mutuelles de Solidarité	MUSO	North Kivu	Community development, health structures, schools, education	Aimed at vulnerable populations in rural areas who do not have access to traditional bank credit	Brings together residents who know and trust each other and decide to jointly solve their problems by financing their project
National poll on perceptions of poverty by the populations of Congo	SOPPOC	North Kivu	Enough food, adequate clothing, shelter, and medical care	Identification of basic needs	Availability of resources addressing basic needs
Association pour la promotion de l'entreprenariat féminin	APEF	South Kivu	Emancipation of women and the redefinition of their place in society	Strengthen the economic activities and social power of women micro-entrepreneurs as well as rural households	Supports low-income women working in the informal sector so that they can increase their livelihoods and improve their economic and social position
Conseil des Organisations Féminines Agissant en Synergie	COFAS	South Kivu	Gender-based violence legal services, health, housing rights and informal settlements, labor and employment, livelihoods, right to information, women's rights	Effective participation of women in the development of South Kivu	Advocacy for gender issues, fighting sexual violence, poverty reduction,<br>legal empowerment, community and reproductive health
Réseau des Femmes pour le Developpement Associatif	RFDA	South Kivu	Training of women in human rights, supporting women who are victims of violence	Identification of victims of sexual violence, training of social workers, social, medical, and legal support	Provides care for 800 women victims of sexual violence in women's houses<br>The network also informs men about women's rights and organizes training on gender, peace, and development.
Centre d'encadrement et d'aide promotionnelle	CEDAP	South Kivu	Promotes the development of communities facing socio-economic and structural vulnerabilities, conflicts, and inter-community and identity tensions	Promotion of peace and sustainable community development	Placing the individual at the center of all actions
Association des Menuiseries de Kihumba	AMEKI	South Kivu, North Kivu	Timber trade and poverty reduction	Poverty alleviation	Fair commercial forestry
Association des Jeunes pour le Développement intégral	AJVDI	North Kivu	Advocacy, conflict, education, environment, food security, health, poverty reduction, youth	Inventory of conflict situations, peaceful conflict resolution, and the promotion of peace	Sharing experiences on the culture of peace
Mining Mineral Resources	MMR	North Kivu	Availability of alternative minerals such as gold or diamonds	Buying minerals from different artisanal mining sites	Availability of canteens where locally produced

					food is available for the miners
Association Rurale des Agriculteurs-Eleveurs de Kiluku	AREAKi	North Kivu	Integration of North Kivu into this vision by capitalizing on its economic and human potentials	Achievement of food security by improving local food production	Reduced poverty of rural populations
Union des Agriculteurs et Eleveurs pour le développement de Kirotshe	UADKi	North Kivu	Capacity-building and institutional support to members, provision of economic services	Support for community and family agro-pastoral production	An economically strong rural population thanks to agro-pastoral activity and the marketing of agricultural and agro-industrial products
Youth Learning Centre	AFIDI	North Kivu	Civic and social organizations	Creating employment opportunities for youth	Access to education for children
Vision d'Appui aux enfants délaissés et orphilins	VAEDO	North Kivu	Health education, water and sanitation, community recovery, and food security	Orphan care	Access to school for orphans
Women's Initiative for Integral Development	IFEDI	North Kivu	Works with "taxi bikers" in Goma	Awareness sessions on violence against women and HIV prevention	By far the most convenient means of transportation (approximately 5,000 throughout the city)
Espérance après violence et abandon	EVA	North Kivu	Offers advice and comfort to victims	To set up exchange platforms in the fight against AIDS and intrafamily violence against women	Support for civil society actors is strengthened.
Groupe d'Hommes Voués au Développement	GHOVODI	North Kivu	Listening houses in which the association's 39 advisers, all volunteers, try to reduce suffering	Improvement in the social and health living conditions of the populations affected by the war in eastern DRC	Existing community fields, where women work together to avoid being isolated and raped on their way to the field
Associations Villageoises d'Epargne et Crédits	AVEC	South Kivu, North Kivu, Ituri Province	Peacebuilding activities	Resilience in the face of risks and unforeseen events, possibility of launching or consolidating income-generating activities	Educating the population on peacebuilding by setting up peace-generating projects such as village savings and credit associations
Association des femmes commerçantes et navigantes	AFCONA	South Kivu, North Kivu	Unity and solidarity between the organizations and their affiliates	To represent the interests of a rather wealthy class of female operators in alliance with smaller cloth traders	More localized or sector-specific organizations
Forum SIDA	FOSI	North Kivu, Ituri Province	Community-based organizations fighting HIV/AIDS and sexual and gender-based violence	Facilitates LGBTQ groups and sex workers to meet on its premises	Raising awareness about HIV/AIDS
Associations Basées sur les Violences	ABV	North Kivu	Psychosocial support and legal advice to confront the perpetrators of violence	Advocate for the protection of civilians against human rights abuses and gender-based violence	Briefing notes to report gender-based violence are produced.
Bureau Central de la Zone de Santé	BCZS	South Kivu, North Kivu, Ituri Province	Implements the primary healthcare strategy	To set up a referral system with community healthcare providers	Availability of the peripheral level, including health posts, health centers, and hospitals of reference
Gender and Development in Practice	GADIP	South Kivu	A network for feminists within academia and civil society	To enhance women's power, economic equality, and peace	Interested and engaged in mutual knowledge and best practice exchange
Precision Agriculture for Development	PAD	South Kivu	Personalized agricultural advice	To support smallholder farmers in developing countries	Providing customized information and services that increase productivity, profitability, and

					environmental sustainability
Action pour la Promotion et la Défense des Droits des Personnes Défavorisées	APPRODEPED	South Kivu	Justice and human rights	To serve all disadvantaged people without distinction of race, sex, ethnicity, or religious belief	Justice and human rights
"Let's unite" in Swahili	Tuungane	South Kivu	Community-driven reconstruction program	To support economic recovery and to improve the quality of local governance and social cohesion	
Parlement des Jeunes		South Kivu, North Kivu, Ituri Province	Made up of young leaders of organizations, associations and movements, religious denominations, and universities.	Building a universe of integrity in favor of a resilient generation	Economic and citizen empowerment
Women together or Women for Development	Mamans Majambere	South Kivu	Meeting rural communities to share information and knowledge on key themes such as HIV/AIDS, violence against women, and food security	Support and coordination of federations of community listeners' clubs in the rural territories	Enables rural communities, especially women, to make their voices heard and benefit from the broadcasts and programs
Association villageoise contre la vulnérabilité		South Kivu	Development, agriculture, livestock, protection, health nutrition, education and training, entrepreneurship and micro-finance, apprenticeship in trades, and other areas of self-help	Promote the well-being of vulnerable people in villages	Men and women have joined village associations established in their villages.
Mkaaji Mpya	MKAAJI	South Kivu	Support family fish farmers and agriculture	Promote primary healthcare and public health	Access to primary healthcare for mothers and children, public and community health education campaigns
Réseau Congolais des Artisans de Paix	RECAP	South Kivu	Peacebuilding	Working on peacebuilding and on the root causes of conflict, as well as the issue of women's leadership	Brings together local organizations working in peacebuilding
Action pour la Lutte Contre l'Ignorance du SIDA	ALCIS	South Kivu	Policies and programs designed and implemented by women sex workers	To promote health and human rights	Cases of physical and sexual violence against sex workers are documented.
Action for Peace and Harmony	APC	South Kivu, North Kivu	Rehabilitation of infrastructure, agriculture, and livestock; awareness of the population about peaceful cohabitation; conflict mediation	Development and conflict transformation	Encourage and support local actors in positive conflict transformation
Groupe d'initiative locale pour le développement intégral	GILDI	South Kivu	Sustainable agriculture, livestock, roads, health, environment, education, and habitat	Promotion of the well-being of the Congolese population	
Comité pour l'Autopromotion à la base	CAB	South Kivu	Support family farming	Promotion of peasant agriculture, with the ambition of establishing agroecological family peasant agriculture	A rural development program that reaches 400,000 inhabitants in a region where eight out of ten households live in poverty
Agency for Sustainable Development Initiatives	AFSDI	South Kivu	Membership fees, donations, and legacies	Create a pilot village that can ensure its self-	Income from self-financing activities

				sufficiency with its own resources	
Mulangane community Radio		South Kivu	Capacity-building producers and awareness of the participation of all in the management	Promoting the rights of forgotten women and girls	Obtain more votes for female candidates for heads of decentralized territorial entities
	ADEMER	South Kivu	Land for the population	Social support in Kahungu	Social support
Association des Taximans Moto de l'Ituri	ATAMOI	Ituri Province	Facilitates travel in areas where taxis are sometimes non-existent	To reduce unemployment, create a mutual insurance company for taxi drivers, and address social issues such as illness, death, etc.	Security in the form of taxi drivers asking for the identity, origin, and destination of their customers before transporting them
Agricultures Committee		Ituri Province	Land-use plans for village territories or simple natural resource management plans	Involve all members of the village community and allow them to engage in decisions on land-use plans, water, and wildlife resources.	Supervision of agricultural practices and sectors. Collective decisions are leading to better governance.
Union of Congolese Patriots	UPC	Ituri Province	A political and militia group in Ituri, formed towards the end of the Second Congo War	IDP learning new skills to prepare them for a life of self-sufficiency	Community members have acquired skills like soap-making and tailoring.
Congolese International Congress	CIC	Ituri Province	Health and environment, human rights, community development, culture, and art	Preparing a new generation of country managers who are much more responsible and capable of restoring the tarnished image of the DRC	Training young people in entrepreneurial spirit and culture, design and develop bankable projects to address youth unemployment and poverty
Action citoyenne contre l'impunité	ACCI	Ituri Province	Interested in the environment, transparency, and the rights of local communities	Human rights	Using national, regional, and international laws as a basis for claim
Association des Taxis-Motos à Bunia	ATMB	Ituri Province	Motorcycle taxis offer advantages compared to classic taxis.	Can help find the criminals who roam day and night through the city of Bunia	Speed and respect for the customer's destination
Land and Physical Environment					
Football pitches		South Kivu, North Kivu, Ituri Province	Accessible to many footballers Engaging football sessions	Sense of belonging	Strengthens communities, reduces loneliness, ethnic harmony, community pride, improvement in social cohesion
Youth spaces or youth clubs		South Kivu	Conduct educational talks around gender issues, hygiene, and awareness-raising	Become involved in local development and fight poverty in the community	Strengthened traditional behavior and customs in the village
Small and big markets		South Kivu, North Kivu, Ituri Province	Guides implementation and works on the root causes of market failures	Promoting sustainable and inclusive development by empowering entrepreneurs	People's income is increased, improved business practices
Churches		South Kivu, North Kivu, Ituri Province	Lay activism, with many lay men and women bearing witness to their faith in the political, economic, and cultural fields	Compensating for the lack of public services through its network of hospitals, social centers, and renowned schools	Appeals for peace in eastern Congo
Radio Bubusa		South Kivu	Capacity building for women as a strategy to tackle rural poverty in Walungu	Ensure that women have access to information, training, and communication	The issue of profound poverty that characterizes the area is addressed.
Guest houses and hotels		South Kivu, North Kivu, Ituri Province	A selection of guest houses and hotels	Tourism	Possibility of accommodation

			available for accommodation		
Beaches		South Kivu, North Kivu	A picturesque and very charming setting	Tourism	Volcanic islands surrounded by beautiful beaches, terraced hills, and fishing villages
Queen Elizabeth house		South Kivu	Tourist site		Tourist site
Center for victims of armed conflicts	CIVIC	South Kivu	Strengthen the capacity of armed actors and relevant institutions during conflict resolution	Support communities affected by conflict	Prevent and respond to civilian harm
Sports clubs for basketball, games rooms for billiards and kicker		South Kivu	Strengthen communities	Sport activities	Reduce loneliness Improve social cohesion
Action Sociale CHECHE	CHECHE	South Kivu	Training the youth who have left school for employment	Fight against poverty, unemployment, and delinquency	Self-financing activities for young people and general services for the proper functioning of the whole constitute reliable aid to the economic development of families and religious communities.
Publishing houses		North Kivu	Print media are almost non-existent outside the capital city.	Free access to information in the public interest	Editorial independence of public broadcasting
Photo studios		North Kivu	Family photo albums, social identities, and cultural values	To preserve and expose the work of Congolese photographers and help define the country in terms other than victimization and conflict	Possibility to archive and preserve the country's history
Réseau communautaire pour la Protection des enfants	RECOPE	South Kivu	Training sessions on the techniques of community action favorable to the prevention of/response to violations of the rights of children	Protection of children against violence	Members of the organization are equipped with the techniques of community action favorable to the prevention of/response to violations of the rights of children and the promotion of peaceful coexistence and non-violence.
Local Institutions					
Primary and secondary schools		South Kivu, North Kivu, Ituri Province	Elementary school is provided.		A certificate of primary and secondary school is awarded.
Centre de Recherche en Science Naturelles	CRSN/Lwiro	South Kivu	Offers studies in biology, geophysics, and the environment	The library of the Natural Sciences Research Center of Lwiro	Provides samples from the region's unique biodiversity.
Agence d'achat des Performances	AAP Santé	South Kivu	To obtain funding from institutional donors and attempt to attain access to human resources	To promote the social contract between the state and communities by ensuring the access of citizens to quality social services through the promotion of the PBF approach	Negotiate and sign the devolution of service agreement public with the Ministry of Health or other donors
Consortium for Improving Agriculture-based Livelihoods in Central Africa	CIALCA	South Kivu	Improving agriculture-based livelihoods through sustainably increased system productivity to enhance income, nutrition security, and the environment	To accelerate the impact of agricultural research for development	Conducting agricultural research for development in Rwanda, Burundi, and eastern Democratic Republic of Congo

Association pour le Développement intégré au Kivu	ADI-Kivu	South Kivu	Farmer–breeders participating in research to address food security issues	Organizes a knowledge exchange session between farmers, breeders, technicians, and agricultural engineers on crop and livestock issues	Family farming is carried out and there is food security.
Solidarité Féminine contre la Pauvreté	SOLIFEM	South Kivu	Development of income-generating activities, especially in the agriculture, livestock, and microcredit sectors.	Female solidarity against poverty	Socio-economic empowerment for marginalized groups
Coopératives d'Epargnes et de Crédit	COOPEC	North Kivu, South Kivu	Microcredits are granted to members by the cooperative.	Social and economic advancement of its members by collecting their savings and granting loans to them.	Entrepreneurship with cooperatives as a new paradigm of struggle against poverty Cooperatives as good opportunities to undertake some business
Institut Supérieur de Développement Rural	ISDR	South Kivu	Socio-economics and planning of rural development or environmentally friendly management of natural resources	Organizing quality education, conducting scientific research, and supporting rural development actions.	A degree in rural development
Institut Supérieur pour la Promotion de la Paix, du Développement et de l'Environnement	ISPDE	South Kivu	To learn how to prevent and positively manage conflicts	Conflict prevention and resolution and promotion of scientific research	Graduates receive on-the-job market training in the field of business management.
Institut Supérieur des Techniques de Développement	ISTD	South Kivu	Offers higher education in community development, the environment, and sustainable development		
Mamas for Africa		South Kivu	Multidisciplinary teams going from village to village every day offer shelter to girls and women in Maison de la Femme.	Mediation for women and female victims of sexual or gender-based abuse	Consolidated presence in rural areas, where the need is greatest
Women for women		South Kivu	Offers women a constructive, dignified way to regain control of their lives	Education, professional preparation, and agro-pastoral	Increased assets for women, engagement with entrepreneurial work, and net earnings; mental health and household diet diversity; and self-confidence and participation in household decision-making and social support
Institut Supérieur Pédagogique	ISP	South Kivu, North Kivu, Ituri Province	Administrative and computer sciences Engineering arts and humanities Exact sciences	Education leading to an advanced diploma	Positive and effective contribution to the socio-economic development of the country
Institut Superieur de Technique Médical	ISTM	South Kivu	Education is offered at higher education institutes for applied medical sciences.	Education leading to an advanced diploma	Graduates could serve the province as teachers and civil society leaders.
Institut Supérieur des Etudes Agronomiques et Vétérinaires	ISEAV	South Kivu	Allow students to specialize in agroforestry, agribusiness, and agricultural entrepreneurship	Education leading to an advanced diploma	Graduates could serve the province as teachers, administrators, and civil society leaders.
Universities	UOB, UEA, UCB, UNP, UNIC, and	South Kivu, North Kivu	Capacity to deliver higher education degrees in the	The ultimate goal is to train students to provide	Increasing access to higher education, potential to improve and save lives

	ULPGL, UNIGOM		country, higher estimated number of students	answers to real-life problems.	
Brasseries, Limonaderies et Malteries	BRALIMA	South Kivu	Production and marketing of beer and soft drinks	To achieve positive social, environmental, and economic impact on the population	Developing different social activities that improve the social life and conditions of the local population
	PHARMAKINA	South Kivu	Industrial extraction of quinine from the harvested bark of the quinquina tree	Production of affordable medicines for poor people	Quinine output is exported as an active pharmaceutical ingredient (API) to pharmaceutical and intermediary product food industries all over the world.
Office Congolais de Cöntrole	OCC	South Kivu	Conformity assessment and verification of aspects of quality, quantity, and price.	Ensure the regularity of commercial transactions and safeguard the interests of the various partners involved in international trade	A response to the need for neutral and independent organizations
Bureau Diocésain des Œuvres Médicales	BDOM	South Kivu	Medical service technique	Contributing to the improvement of the state of health of the population in South Kivu	
Office des routes	OR	South Kivu, North Kivu, Ituri Province	Road infrastructure works quality control of building materials Promotion of new construction technologies	To manage the network of roads of general interest	Development, construction, and modernization of infrastructures
Société Nationale d'Assurances	SONAS	South Kivu, North Kivu, Ituri Province	To stimulate capital and promote savings	Selling insurance and settling claims	To finance the national economy
Société nationale de chemin de fer du Congo	SNCC	South Kivu, North Kivu, Ituri Province	Commercial company responsible for public transport	Public transport	Railways, lakes, roads, and rivers
Children's Village Bukavu	SOS	South Kivu	Social center, small loans, food packages and medicine, kindergarten and schooling for children	To strengthen vulnerable families in Bukavu and help them to become self-sufficient	Strengthen vulnerable families and help them to become self-sufficient;, enhance the parent's or caretakers' chance of finding a job, enable parents to attend their jobs knowing that their children are well cared for, and help children and their families to achieve a brighter future in the long-term
Actions Collectives pour le Développement Social	ACODES	North Kivu	Works to improve the socio-economic, health, and environmental living conditions of disadvantaged local populations	To encourage environmentally friendly values and behaviors and to teach sustainable fishing practices	Promotion of human rights, gender equality, economic empowerment, education, child protection, environmental protection, and habitat and land protection
Microfinance Institutions and Savings and credit cooperatives	MFIs and SACCOS	North Kivu	Service a million accounts in the province	Financial inclusion by helping the poorest populations to obtain access to basic financial services, also in rural areas.	Local economic growth, provision of mid- to long-term financing to financial institutions
City Hall		South Kivu, North Kivu, Ituri Province	Each town hall brings together municipalities.	Management of a city	Each city is headed by a mayor.
Hospitals		South Kivu, North Kivu, Ituri Province	Over 401 hospitals and clinics in the country	To organize more specialized treatment	Early childhood vaccinations, maternal care, as well as treatment of chronic diseases
Individuals					

Elected members of parliament	MP	South Kivu, North Kivu, Ituri Province	List of candidates from which voters can select their favorite	To ensure the proper functioning of new institutions	Elected through a proportional electoral system for five-year terms
Nurses and midwives		South Kivu, North Kivu, Ituri Province	Ability to help vulnerable populations	Evidence-based healthcare to fit local conditions and necessities	Serve the country as healthcare professionals
Medical doctors	MD	South Kivu, North Kivu, Ituri Province	Ability to help vulnerable populations	Evidence-based healthcare to fit local conditions and necessities	Serve the country as healthcare professionals
Engineers		South Kivu, North Kivu, Ituri Province	They hold a degree from a faculty of law after 5 years of study and have jurisdiction all over the country		Involved in architectural, engineering, and other technical activities
Lawyers		South Kivu, North Kivu, Ituri Province	Offers legal support in different aspects, including commerce, mining, energy, food, agro-business industry, safety and security, and environmental issues and biodiversity	Promotion of justice and equal opportunities for all citizens, as well as defense of human rights	Meet the needs of the civilians who require legal assistance
Priests		South Kivu, North Kivu, Ituri Province	Diocesan priests ministering in parishes and dioceses	Lay activism and its widespread presence in society and in the media	The lack of public services is compensated for through the network managed by priests.
Master tailors		South Kivu, North Kivu, Ituri Province	Blouses, dresses, shirts, and headscarves come in every color, such as maraschino cherry.	To provide the population with training leading to professional certification and income-generating activity.	Population able to start their own businesses and have income
Traditional chiefs		South Kivu, North Kivu, Ituri Province	Traditional chiefs are co-opted by the provincial deputies on the basis of one chief for each territory.	Educate traditional chiefs on their role in the fight against impunity for sexual violence	Trained in human rights and well-informed about counseling services for victims of sexual violence.
Traditional healers		South Kivu, North Kivu, Ituri Province	Combine modern medicine with traditional healing	Offering conventional and herbal treatments	Many of the herbs used are gathered by local indigenous groups, who historically lived in forests and have vast knowledge about local plants and herbs.
Musicians		South Kivu, North Kivu, Ituri Province	People have long loved, cried, lived, and danced to the rhythms of Congolese beats.	To explore possible linkages with civil society and established media outlets, to offer a model of leadership by using music as a public voice	Congolese music distracts and entertains the hapless masses.
Teachers		South Kivu, North Kivu, Ituri Province	Context, teacher participation, and supportive community building	To improve the low level of learning standards	More children who start primary school complete the basic education cycle
Community leaders		South Kivu, North Kivu, Ituri Province	Social cohesion and peaceful coexistence between communities	Promoting peaceful coexistence among all communities	Community leaders signed a joint statement that was used to further disseminate their message.
Artisanal mining	Mines	South Kivu, North Kivu, Ituri Province	Driver of development in communities where there are often few other opportunities for generating income	Contributes to many of the Sustainable Development Goals	With comprehensive formalization, the global community can mitigate negative impacts.
Plantations IRABATA		South Kivu	Approximately 43% of the plantation area was unexploited.	Exploiting the tea plant, Cinchona, afforestation	Tea factory, Cinchona, afforestation

Owner of a health center	North Kivu	Service delivery, access to essential medicines, and health information systems	Access to basic healthcare services	Improved access to healthcare in terms of volume and quality for the local community
Plantation GOMBO	South Kivu	Okra has 25 workers distributed in different areas, and 20 people are working in the tea factory.	Food security—fight against erosion, agroforestry, the production of fertilizers by composting plant waste and animal excrement, marsh drainage, cultivation on ridges	Production from the Okra plantation is oriented towards individual consumption.
Community workers	South Kivu, North Kivu, Ituri Province	Plumbers, masons, carpenters, traders		
Colonels	Ituri Province	Campaigning, sharing intelligence with the army, and maintaining a sufficient presence	Maintaining civil order during tense times	Fewer kidnappings, fewer civilians are killed, and fewer attacks on civilians
Culture, History, and Stories				
Cultural center of Goma	North Kivu	Training programs in painting, sculpture, carving, architecture, and ceramics, as well as traditional music and drama.	Promotion of cultural life and arts	Artists write poetry, plays, and novels in local languages.
Cultural center of Ituri	Ituri Province	Organizing festivals of local artists, film projections, etc.	Promotion of cultural life and arts	
Cultural center of Bukavu	South Kivu	Organizing festivals of local artists, film projections, etc.	Conflict management and the promotion of peace	
Movie room	South Kivu, North Kivu, Ituri Province	Film projections	To channel people's energy into arts, not conflict	The weary victims of war have a chance, a moment, to forget their misery.
Local Economy and Exchange				
Local product from the nearest city of Bukavu	South Kivu	Offers green shoots of hope to local communities and facilitates greater "agri-preneurship" among local youth, thereby contributing to stabilization and economic recovery in some of the most fragile and conflict-affected areas in the country	To achieve security in sustainable food	A local entrepreneur has employed more youths to increase the production of its cookies and biscuits.
Breeding	South Kivu, North Kivu	Plants, animals, fertile country with approximately 80 million hectares of arable land	To improve food insecurity issues in the long term	Smallholder farmers are practicing family farming market-oriented agriculture and reducing gender inequalities while empowering women.
Craft sector	South Kivu, North Kivu	Basketry, sewing, carpentry, bricks, etc.	Oversight of the development of non-agricultural activities, including crafts	The craft sector is expanding and well-developed.
Agriculture	South Kivu, North Kivu	Local products such as cassava, banana, bean, coffee, and groundnut	To develop the agricultural sector and strengthen its contribution to economic growth, restore the country's food security, and reduce poverty and insecurity in rural areas	Marketing of harvest products

Livestock and agro-pastoral		South Kivu, North Kivu	The largest livestock farming region in the country	To contribute to the achievement of food security	Improved access to markets and value-added agricultural products
Fishing and maritime traffic		South Kivu, North Kivu, Ituri Province	Observation of maritime distress situations, naval exercises, war situations, locations of sinking ships and drifting vessels, and salvage of crews and passengers		
Man Day Labor Sale		South Kivu	Three key sectors with a large part of the workforce: agriculture, construction, and mining	Promote job creation	Transfer of labor from low-productivity sectors to productive sectors
Small subsistence shops, stores		South Kivu, North Kivu	The majority of foodstuff in the country is still purchased in the traditional system.	To improve the food security of the most vulnerable populations	Traditional shops maintain their prominent role, making up more than half of the market.
Soap factories		South Kivu	Detergents, edible oils, margarine, and cosmetics	To promote and transform local production into finished products for the local economy	Local products everywhere
Electronic repair		South Kivu	Pick-up and delivery service	Electrical repair and maintenance	People are assisted—no need to purchase new devices
Sale of services		South Kivu, North Kivu	Priorities include (i) good governance and consolidating peace, (ii) macroeconomic stability and economic growth, (iii) access to social services, and (iv) promoting community dynamics.	To increase the utilization of effective services	Transport, secretariat, organizers of events
Trade and other businesses		North Kivu	The region is home to industrial and artisanal mines extracting cobalt, gold, and diamonds, as well as a rich agricultural sector.	To improve the business climate and increase trade investment	Import and export potential
PREMIDIS SARL		North Kivu	Construction of schools, markets, monuments, state administrative buildings, roads, bridges, universities, etc.)	Involved in several social actions	Partner of the City Hall, financing the construction of the office of the urban police station and a pavilion of a Catholic university
Société Aurifère du Kivu et du Maniema	SAKIMA	South Kivu, North Kivu	Owns hydroelectric power stations and various workshops to support mining production	State-owned mining company that holds interests in various gold and tin mines in the eastern Congo	
Nyiragongo Ciment		North Kivu	Cement production	Plant-based cement production	Produce quality cement
Farms		North Kivu	About 70% of the employed population is engaged in agriculture, mostly for subsistence.	To promote inclusive economic growth, reduce poverty, and enhance food security	Food security of vulnerable households, durable peace, and improved nutritional uptake activities
Artisanal mining		South Kivu, North Kivu	Mainly for gold, cassiterite, coltan, diamonds, tourmaline, pyrochlore, and wolfram	To comply with the requirements of transparency and traceability	Agreements and sorting out of differences among all stakeholders
Sale of market garden products		South Kivu, North Kivu	Favorable pedoclimatic conditions for different types of crops, such as market gardening, which	Characterization of market gardening systems and assessment of existing systems and perspectives to	Growing market gardening in the agricultural production system of the province

		is one of the evolving agricultural sectors within the province.	enable a steady transition to integrated, sustainable, and resilient crop systems	
Travel agents	North Kivu, South Kivu	The Congo River, gorilla tours, the Congo Rainforest, and seeing the animals, tribes, and culture	To provide real, guided, safe, comfortable vacations in the Congo.	Tourism
Bakeries	South Kivu, North Kivu	A good choice and sandwiches that are always fresh	To produce flour-based products that meet the expectations of the local consumers	They sell bread, several kinds of pastries, and cakes.
Parking for motorcycles	Ituri Province	Parking areas and roads with high volumes of movement.	Increase access to hard-to-reach areas	Offers a mode of transportation, the possibility to wait for customers in the parking lot
Local markets	Ituri Province	Locally produced commodities are traded over short distances.	To improve access to food and water	People work in fields and earn a little money in trade at the local market.
Food shops	Ituri Province	A new agriculture revival initiative to grow more food close to home.	Resources needed for basic healthcare and assistance	Improvements in food security, reduced need for nutritional support
